# Impact of Genetically Predicted Red Blood Cell Traits on Venous Thromboembolism: Multivariable Mendelian Randomization Study Using UK Biobank

**DOI:** 10.1161/JAHA.120.016771

**Published:** 2020-07-08

**Authors:** Shan Luo, Shiu Lun Au Yeung, Verena Zuber, Stephen Burgess, Catherine Mary Schooling

**Affiliations:** ^1^ School of Public Health Li Ka Shing Faculty of Medicine The University of Hong Kong Hong Kong SAR, China; ^2^ Medical Research Council Biostatistics Unit School of Clinical Medicine University of Cambridge United Kingdom; ^3^ Department of Epidemiology and Biostatistics Imperial College London London United Kingdom; ^4^ Medical Research Council/ British Heart Foundation Cardiovascular Epidemiology Unit School of Clinical Medicine University of Cambridge United Kingdom; ^5^ School of Public Health and Health Policy City University of New York NY

**Keywords:** hemoglobin, mendelian randomization, venous thromboembolism, Epidemiology, Thrombosis, Genetics

## Abstract

**Background:**

Red blood cell (RBC) transfusion and erythropoiesis‐stimulating agent administration are cornerstones of clinical practice, yet concerns exist as to potential increased risk of thrombotic events. This study aims to identify RBC traits most relevant to venous thromboembolism (VTE) and assess their genetically predicted effects on VTE in the general population.

**Methods and Results:**

We used multivariable mendelian randomization with bayesian model averaging for exposure selection. We obtained genetic variants predicting any of 12 RBC traits from the largest genome‐wide association study of hematological traits (173 480 participants of European ancestry) and applied them to the UK Biobank (265 424 white British participants). We used univariable mendelian randomization methods as sensitivity analyses for validation. Among 265 424 unrelated participants in the UK Biobank, there were 9752 cases of VTE (4490 men and 5262 women). Hemoglobin was selected as the plausible important RBC trait for VTE (marginal inclusion probability=0.91). The best‐fitting model across all RBC traits contained hemoglobin only (posterior probability=0.46). Using the inverse variance–weighted method, genetically predicted hemoglobin was positively associated (odds ratio, 1.21 per g/dL unit of hemoglobin; 95% CI, 1.05–1.41) with VTE. Sensitivity analyses (mendelian randomization–Egger, weighted median, and mendelian randomization pleiotropy residual sum and outlier test) gave consistent estimates.

**Conclusions:**

Endogenous hemoglobin is the key RBC trait causing VTE, with a detrimental effect in the general population on VTE. Given men have higher hemoglobin than women, this finding may help explain the sexual disparity in VTE rates. The benefits of therapies and other factors that raise hemoglobin need to be weighed against their risks.

**Nonstandard Abbreviations and Acronyms**
ESA: erythropoiesis‐stimulating agent
GWAS: genome‐wide association study
*ICD‐9*: *International Classification of Diseases, Ninth Revision*
*ICD‐10*: *International Classification of Diseases, Tenth Revision*
IVW: inverse variance weighted
MAF: minor allele frequency
MCH: mean corpuscular hemoglobin
MIP: marginal inclusion probability
MR: Mendelian randomization
MR‐BMA: multivariable mendelian randomization based on bayesian model averaging
MR‐PRESSO: mendelian randomization pleiotropy residual sum and outlier test
PP: posterior probability
RBC: red blood cell
VTE: venous thromboembolism

**Clinical Perspective**

**What Is New?**

- Red blood cell attributes are highly correlated genetically and phenotypically, making it difficult to disentangle causal drivers of disease.
- This study used multivariable mendelian randomization with bayesian model averaging to select and prioritize between 12 red blood cell traits, which suggested endogenous hemoglobin is the key factor for venous thromboembolism, with a detrimental effect in the general population.

**What Are the Clinical Implications?**

- This study is consistent with randomized controlled trials showing that increasing hemoglobin in patients with anemia, via blood products, erythropoiesis‐stimulating agents, or blood transfusion, increases the risk of thromboembolic events.
- This study also suggests relevance to the general population as well as to patients.
- The benefits of therapies and other factors that raise hemoglobin need to be weighed against their risks.

Red blood cell (RBC) transfusion is the most readily available method to alleviate anemia and bleeding resulting from a variety of clinical conditions, yet concerns exist as to the risk of adverse effects, with several trials ongoing to establish the optimal transfusion threshold in patients.^1^ Erythropoiesis‐stimulating agents (ESAs) are widely used in clinical practise to increase hemoglobin concentration by mimicking endogenous erythropoietin and stimulating erythropoiesis in the bone marrow in response to cellular hypoxia.^2^ Systematic reviews and meta‐analyses of randomized controlled trials of ESAs for treatment of anemia in patients have found increased risk of thrombotic vascular events.^3^, ^4^ In 2007, the US Food and Drug Administration issued a public health advisory about the increased risk on ESAs of blood clots and venous thromboembolism (VTE), and required a label warning suggesting more caution when using ESAs,^5^ as reflected in recent clinical practice guidelines.^6^ Notably, similar warnings have also been issued about specific ESAs,^7^ which induce erythropoiesis^8^ and may drive the association with VTE.^9^ Correspondingly, targeting lower erythroid cells for polycythemia vera reduced the rate of major thrombosis.^10^ However, it is unclear whether these findings are the result of specific interventions in patients, and whether they extend to the general population across the normal range is yet to be determined.

Several observational studies have assessed the role of RBC attributes in thrombosis in the general population.^11^, ^12^ A study of hemoglobin concentration and its changes in healthy young women to avoid bias from confounding by ill health and selection bias suggested hemoglobin concentration increased thrombosis.^11^ Several RBC attributes are altered simultaneously by RBC transfusion and ESAs, and RBC attributes are highly correlated both genetically and phenotypically,^13^ making it difficult to disentangle the causal drivers of disease risk. As RBC transfusion and ESA administration are cornerstones of clinical practice, better understanding of the causal determinants of thrombosis has critical clinical importance and public health implications in the general population.

Mendelian randomization (MR), using genetic variants randomly allocated during conception as instrumental variables, is less prone to confounding than traditional observational studies, and can help ascertain causal effects.^14^ MR, at the interface of experimental and observational studies, provides a distinct strand of genetic evidence on potential targets of interventions. Multivariable MR models multiple exposures simultaneously, accounting for measured pleiotropic effects via any of the observed exposures.^15^ Previous studies have used univariable and multivariable approaches to assess the effects of genetically predicted blood cell traits on disease risk^13^, ^16^; however, these analyses have been limited in statistical power and their ability to consider high‐dimensional highly correlated attributes, such as all 12 RBC traits. To address these limitations, we used a novel approach for multivariable MR based on bayesian model averaging (MR‐BMA), which scales to the high‐throughput candidate exposures and enables exposure prioritization in a bayesian framework.^17^ MR‐BMA performs well even when the exposures considered are highly correlated because of biological processes.^17^ In the UK Biobank, we used MR‐BMA to select the RBC traits most relevant to VTE both on average and individually. We then assessed the effects of the top‐ranking exposure(s) on VTE in univariable MR.

## Methods

The UK Biobank received ethical approval from the research ethics committee (11/NW/0382), and participants provided written informed consent. Summary statistics were generated from publicly available data that had previously received appropriate ethics and institutional review board approvals, and further sanction was therefore not required. The individual‐level data in the UK Biobank are available by application directly to the UK Biobank. The data that support the findings of this study are available from the corresponding author on reasonable request. The statistical code in R for implementing MR‐BMA can be obtained from the open‐source code from Github (https://github.com/verena-zuber/demo_AMD).

### Study Design

This is a 2‐sample multivariable MR study, which relies on 3 instrumental variable assumptions (Figure S1). First, the genetic variant is associated with at least one of the exposures. Second, the variant is independent of all confounders of each of the exposure‐outcome associations. Third, the variant is independent of the outcome conditional on the exposures and confounders.

### Genetic Predictors of Endogenous RBC Traits

Genetic predictors of RBC traits were extracted from summary statistics generated from an existing publicly available genome‐wide association study (GWAS) of hematological traits conducted in 173 480 participants of European ancestry without any blood cancer or other major blood disorder.^13^ Participants were from the UK Biobank (132 959, 52% women) and the INTERVAL (Efficiency and safety of varying the frequency of whole blood donation) studies (40 521, 50% women).^18^ Blood samples for full blood count analysis were collected by venipuncture in EDTA tubes, and measured by a combination of fluorescence and impedance flow cytometry at the centralized processing laboratory of UK Biocentre (Stockport, UK) within 36 hours.^13^ Genotyping was undertaken with Affymetrix Axiom 2.0 Array, and variants were excluded if they deviated from Hardy‐Weinberg equilibrium (*P*<5×10^−6^), the within‐batch call rate was <97%, the across‐batch call rate was <75%, or they were nonautosomal biallelic.^13^ Imputation was performed using a combined 1000 Genomes phase 3 and UK 10K imputation panel.^13^ Univariable associations of each RBC trait with 29.5 million variants (with imputation information score >0.4 and minor allele frequency [MAF] >0.01%) were obtained from linear mixed model using BOLT‐LMM v2.2,^19^ adjusted for the top 10 principal components of ancestry and adjusted for recruitment center.^13^

### Selection of Genetic Variants

We obtained genetic variants that robustly (genome‐wide significance *P*<8.31×10^−9^, a recent threshold for genome‐wide analyses of common, low‐frequency, and rare variants) and independently (*r* ^2^<0.001) predicted any of the 12 RBC traits (ie, RBC count, mean corpuscular volume, hematocrit, hemoglobin concentration, mean corpuscular hemoglobin [MCH], MCH concentration, red cell distribution width, reticulocyte count, reticulocyte fraction of red cells, immature fraction of reticulocytes, high light scatter reticulocyte count, and high light scatter reticulocyte percentage of red cells). These variants were checked for imputation quality and validity as instrumental variables using individual data from the UK Biobank, with the following exclusion criteria: (1) imputation information score <0.3 for MAF >3%, information score <0.6 for MAF 1% to 3%, information score <0.8 for MAF 0.5% to 1%, and information score <0.9 for MAF 0.1% to 0.5%; (2) departure from Hardy‐Weinberg equilibrium at Bonferroni‐corrected significance; (3) associated with potential confounders (described below) of the variant‐outcome relation at Bonferroni‐corrected significance; (4) in the *ABO* gene, which is well known to be highly pleiotropic;^20^ or (5) were equivocally palindromic (allele frequency close to 0.5).

### Genetic Association With VTE

The UK Biobank recruited ≈500 000 participants intended to be aged 40 to 69 years from 2006 to 2010 at 22 recruitment centers across Scotland, Wales, and England in the United Kingdom.^21^ Participants provided samples, completed questionnaires, including self‐reported diseases and regular prescription medications, underwent assessments, and had nurse‐led interviews. Longitudinal follow‐up via record linkage to all health service encounters and deaths is ongoing. Prevalent and incident diseases were defined using *International Classification of Diseases, Ninth Revision* (*ICD‐9*), and *International Classification of Diseases, Tenth Revision* (*ICD‐10*), codes. Causes of death were classified using *ICD‐10* codes. Genotyping was undertaken with 2 similar arrays, the UK Biobank Lung Exome Variant Evaluation Axiom array (49 979 participants) and the UK Biobank Axiom array (438 398 participants).^21^ Genotype imputation was to a reference set combining the UK10K haplotype and the Haplotype Reference Consortium reference panels.^21^ To reduce confounding by a hereditary tendency to thrombophilia^22^ and latent population structure,^23^ we restricted the analysis to genetically verified white British participants and further excluded participants with (1) withdrawn consent, (2) sex mismatch (genetic sex differs from reported sex), (3) aneuploidy of sex chromosomes, (4) low‐quality genotyping (missing rate >1.5%), or (5) relatedness (greater than putative third‐degree relatives in the kinship table).^21^ We used genotype and phenotype data from the UK Biobank provided in March 7 and November 6, 2018, updates.

### Exposures

The exposures were 12 genetically predicted RBC traits (ie, RBC, mean corpuscular volume, hematocrit, hemoglobin, MCH, MCH concentration, red cell distribution width, reticulocyte count, reticulocyte fraction of red cells, immature fraction of reticulocytes, high light scatter reticulocyte count, and high light scatter reticulocyte percentage of red cells).

### Outcome

We developed classification algorithms for VTE following the recommendations of the UK Biobank.^24^ We defined VTE on the basis of self‐report at baseline (internal UK Biobank codes 1068, 1093, and 1094) or subsequent primary or secondary diagnosis of hospital episodes (*ICD‐9* 415.1, 416.2, and 451–453 and *ICD‐10* I26 and I80–I82) or underlying and contributory causes of death (*ICD‐10* I26 and I80–I82). Incident and prevalent cases of VTE were combined to maximize statistical power, under the implicit assumption that all events occur incident to a genetic exposure.

### Potential Confounders

To check the randomization, we assessed the association of each genetic variant with potential confounders (ie, established risk factors substantially affecting both hematological traits^13^ and higher risk of VTE^25^) in the UK Biobank. Body mass index was calculated as weight divided by height squared (kg/m^2^). Smoking and alcohol drinking status were categorized as never, previous, current smoker/drinker, and prefer not to answer. Educational level was categorized into degree/professional, nondegree, none of the above, and prefer not to answer, derived from the questionnaire. Townsend deprivation index (a composite indicator of socioeconomic status) was based on preceding census data for area of residential postcode at the baseline visit.

### Statistical Analysis

Analysis of variance (continuous) and χ^2^ tests (categorical) were used to assess whether each genetic variant was associated with the potential confounders. The association of each variant with VTE was obtained using an additive genetic model, adjusted for sex, age, genotyping array, and 40 principal components of genetic ancestry.

### Multivariable MR Based on Bayesian Model Averaging

Exposure selection was performed using MR‐BMA. On the assumption that one of the models considered is true, MR‐BMA ranks all these submodels from the larger model where all 12 RBC traits could have a causal effect on VTE (ie, a single RBC trait or a combination of multiple RBC traits on VTE),^15^ according to the posterior probability (PP) of their associations with the outcome. PP is the probability, given the larger model and a set of priors, that a submodel is true. PP is derived from a bayesian model fit criterion, which assesses how well a linear combination of genetic associations with RBC traits predicts the genetic associations with VTE. To aggregate the evidence for individual trait, we combine evidence across all models that include the particular RBC trait(s). The marginal inclusion probability (MIP) is the sum of the PP over all models, including the RBC trait. Outliers were quantified by Q statistic, and influential variants were identified by Cook distance. We repeated the analyses excluding outliers (Q >10) or influential variants (d > median variant of the relevant F‐distribution) consistently detected in all the best models (PP >0.02). The flow of MR‐BMA is depicted in the Figure.

As recommended,^15^ with 12 RBC traits, we initially set prior probability *P*=0.1, corresponding to a priori expecting 1.2 causal factor (p×d). On the basis of a simulation study,^15^ we fixed the prior variance δ^2^=0.25, corresponding to the priori for the variance of RBC traits. To check the impact of the prior selection, we varied the prior probability of selecting a causal factor from *P*=0.2 to 0.4, reflecting 2.4 to 4.8 expected causal factors.

We also excluded the top‐ranking exposure from each model to check if any alternative exposure had equally strong probability of causality. Finally, as platelets may play a role in the development of VTE, we additionally included 4 platelet traits (platelet count, mean platelet volume, platelet distribution width, and plateletcrit) as alternative potential exposures to assess if these play a role.

### Sensitivity Analyses

To verify our finding from MR‐BMA, we used several univariable MR methods. We used an inverse variance–weighted (IVW) multiplicative random effects meta‐analysis of the genetic variant–specific Wald estimates. IVW provides unbiased estimates as long as all genetic variants are valid instruments. The weighted median provides valid estimates if at least 50% of the weight comes from valid variants.^26^ MR‐Egger is an extension of IVW but captures horizontal pleiotropy as long as the instrument strength is independent of the direct effect.^27^ The MR‐Egger intercept, with *P*<0.05, indicates presence of a pleiotropic effect, suggesting the IVW estimate is invalid. MR‐Egger can have low statistical power, so we concentrated on the direction and effect size rather than statistical significance. MR pleiotropy residual sum and outlier test (MR‐PRESSO), which assumes instrument strength is independent of the direct effect and at least 50% of the variants are valid, is a statistical method for detecting and, if necessary, correcting for horizontal pleiotropic outliers.^28^ An MR‐PRESSO global test *P*<0.05 (on the basis of 10 000 simulations) indicates horizontal pleiotropy. The MR‐PRESSO uses the difference between the observed and expected distribution of RSS residual sum of squares for each variant to identify potentially horizontal pleiotropic outliers, and provides a corrected estimate by removing these outliers.^28^ Finally, we excluded any variants associated with cholesterol and/or ischemic heart disease identified from PhenoScanner (*P*<5×10^−8^).^29^

In 2‐sample MR, sample overlap may introduce bias and inflate type I error rate, when weak instrument bias is present.^30^ The bias depends on the proportion of overlap and the instrument strength.^30^ The bias can be estimated as the product of the bias of the observational estimate, the proportion of overlap, and the reciprocal of instrument strength.^30^ As the hematological GWAS included 132 959 participants who were randomly selected from the UK Biobank, we estimated the bias using an online tool (https://sb452.shinyapps.io/overlap/).

Genetic associations with the outcome were estimated using the *SNPTEST v2.5.4* program. MR‐BMA was performed using the open‐source code from Github (https://github.com/verena-zuber/demo_AMD). Univariable MR analyses were performed using the *TwoSampleMR* and *MR‐PRESSO* packages in the R version 3.4.4 software platform (R Development Core Team, Vienna, Austria). Two‐sided *P* values are reported throughout.

## Results

Of the 731 genetic variants independently predicting any of the 12 RBC traits at genome‐wide significance, 648 remained after excluding on imputation quality, Hardy‐Weinberg equilibrium, association with potential confounders, being in the *ABO* gene, or being equivocally palindromic (Figure S2). After applying extensive exclusion criteria, the mean age of 265 424 unrelated participants (123 809 men and 141 615 women) was 56.9 years, with 9752 cases of VTE (4490 men and 5262 women) used in the analysis.

When including all genetic variants available for the RBC traits (n=648), the exposure most relevant to VTE on the basis of MIP was hemoglobin (MIP=0.90); all other RBC traits had MIP <0.24 (Table S1). To check model fit, we used the best individual models with PP >0.02 (Table S1). Seven outlying variants were identified with high Q statistics (Q >10) consistently in these best models (Table S2 and Figure S3). No influential variant was identified by Cook distance (Table S3 and Figure S3).

We repeated the analysis without the 7 outlying variants (n=641). Again, the most relevant RBC trait was hemoglobin (MIP=0.91), which was followed in relevance by hematocrit (MIP=0.28) (Table 1). Genetic associations with hemoglobin and hematocrit were strongly correlated (*r*=0.91), and models including both had relatively low probability (PP=0.09; Table 2). Figure S4 shows the scatterplots of the genetic associations with each of hemoglobin and hematocrit individually against the genetic associations with VTE risk. We selected the 5 best individual models with PP >0.02 and verified the model fit (Figure S5); no variant with consistently large Q statistics or Cook distance was observed (Tables S4 and S5). We tested the robustness of the results with respect to different initial prior probability parameters that did not alter the ranking of RBC traits (Table S6). As a further sensitivity analysis, we repeated the analysis with several sets of RBC traits. Hemoglobin was still selected with the highest MIP when removing highly correlated hematocrit. MCH (ie, hemoglobin/RBC) was the top exposure when hemoglobin was removed (Table S7). Hemoglobin was also selected with the highest MIP when also considering platelet traits. All of which suggest the effect of hemoglobin is insensitive to the specific selection of RBC traits.

**Table 1:** Ranking of RBC Traits According to Their MIP for VTE in the UK Biobank After Exclusion of Outlying Variants (n=641) Using MR‐BMA

	Exposure	MIP	Model‐Averaged Causal Estimate (OR)
1	Hemoglobin	0.912	1.22
2	Hematocrit	0.275	0.95
3	HLSR	0.154	0.98
4	MCHC	0.108	1.01
5	RET%	0.104	0.99
6	IRF	0.084	1.01
7	HLSR%	0.076	1.00
8	RET	0.070	1.00
9	RBC	0.067	1.00
10	MCH	0.060	1.01

HLSR indicates high light scatter reticulocyte count; HLSR%, high light scatter reticulocyte fraction of red cells; IRF, immature fraction of reticulocytes; MCH, mean corpuscular hemoglobin; MCHC, MCH concentration; MIP, marginal inclusion probability; MR‐BMA, multivariable mendelian randomization based on bayesian model averaging; OR, odds ratio; RBC, red blood cell; RET, reticulocyte count; RET%, reticulocyte fraction of red cells; and VTE, venous thromboembolism.

**Table 2:** Ranking of Models (ie, Sets of Exposures) According to Their PP for VTE in the UK Biobank After Exclusion of Outlying Variants (n=641) Using MR‐BMA

	Exposure(s)	PP	Model‐Specific Causal Estimate (OR)
1	Hemoglobin	0.461	1.16
2	Hematocrit, hemoglobin	0.085	0.82, 1.38
3	Hemoglobin, HLSR	0.034	1.19, 0.94
4	Hematocrit, hemoglobin, HLSR	0.030	0.77, 1.52, 0.92
5	Hemoglobin, RBC	0.024	1.21, 0.94
6	Hemoglobin, MCHC	0.019	1.14, 1.07
7	Hematocrit, hemoglobin, RET%	0.018	0.74, 1.54, 0.93
8	MCH, RBC	0.017	1.15, 1.16
9	Hemoglobin, MCH	0.017	1.14, 1.04
10	Hematocrit, hemoglobin, HLSR%, IRF	0.015	0.67, 1.73, 0.81, 1.23

HLSR indicates high light scatter reticulocyte count; HLSR%, high light scatter reticulocyte fraction of red cells; IRF, immature fraction of reticulocytes; MCH, mean corpuscular hemoglobin; MCHC, MCH concentration; MR‐BMA, multivariable mendelian randomization based on bayesian model averaging; OR, odds ratio; PP, posterior probability; RBC, red blood cell; RET%, reticulocyte fraction of red cells; and VTE, venous thromboembolism.

Estimates for hemoglobin using univariable MR are shown in Table 3 on the basis of 81 and 72 variants (after exclusion for known potential pleiotropy) (Table S8). Using IVW, hemoglobin was consistently positively associated with VTE. The weighted median, MR‐Egger, and MR‐PRESSO estimates were of similar magnitude and were also directionally consistent (Table 3), suggesting that bias caused by horizontal pleiotropy is unlikely, assuming the genetic instruments do not directly affect a confounder of RBCs on VTE.

**Table 3:** Effect of Genetically Predicted Hemoglobin Concentration on the Risk of VTE in the UK Biobank Using Univariable MR

Variant*	Method	OR (95% CI)	*P* Value	Intercept	*P* Value^†^	*P* Value^‡^
81	IVW	1.21 (1.05–1.41)	0.01			
	MR‐PRESSO	1.21 (1.06–1.38)	0.01			<0.001
	MR‐Egger	1.39 (1.04–1.87)	0.03	−0.006	0.30	
	Weighted median	1.18 (0.99–1.41)	0.06			
72	IVW	1.20 (1.05–1.37)	0.01			
	MR‐PRESSO	No significant outliers				
	Weighted median	1.18 (1.00–1.40)	0.05			
	MR‐Egger	1.28 (0.98–1.67)	0.08	−0.003	0.59	

IVW indicates inverse variance weighted; MR, mendelian randomization; MR‐PRESSO, MR pleiotropy residual sum and outlier test; OR, odds ratio; and VTE, venous thromboembolism.

* Variant indicates number of genetic variants.

^†^ *P* value for MR‐Egger intercept.

^‡^ *P* value for global test, indicates horizontal pleiotropy.

## Discussion

This MR study using MR‐BMA to choose between 12 correlated RBC traits suggests hemoglobin is the RBC trait most relevant to VTE. This finding is consistent with randomized controlled trials in patients showing that increasing hemoglobin in anemia, via blood products, ESAs, or blood transfusion, increases thromboembolic events.^31^ Our study also suggests relevance to the general population as well as previously seen in patients.

Hemoglobin is a functional protein released from RBCs into the circulation when RBCs are removed by phagocytic activity or hemolysis. Increases in total intracellular hemoglobin or excessive extracellular hemoglobin in chronic and acute anemia can clog blood vessels.^32^ Experimental evidence shows hemoglobin and associated stasis augment platelet adhesion reactivity (a well‐established coagulator) in vivo and in vitro, even with a low platelet count, independent of hematocrit.^33^ Hemoglobin inhibits the a disintegrin and metalloproteinase with a thrombospondin type 1 motif, member 13, cleavage of von Willebrand factor proteolysis.^34^ Increasing a disintegrin and metalloproteinase with a thrombospondin type 1 motif, member 13, activity reduces ischemic heart disease,^35^ but effects on VTE have not been assessed. Hemoglobin also increases endothelin‐1 to rapidly and irreversibly scavenge NO, favoring systemic vasoconstriction and platelet activation, creating conditions that lead to intravascular thrombosis.^36^ Our study suggests higher endogenous hemoglobin protein in RBCs relates to thromboembolic events, assessed from hemoglobin or from hematocrit as the ratio of the number of RBCs/total blood cells. Further investigation of the mechanistic role of hemoglobin protein in thrombosis is warranted.

The main strength of this study is the implementation of MR‐BMA to select and prioritize potential drivers of VTE from 12 RBC traits, accounting for widespread pleiotropy of highly correlated RBC traits, with validation and provision of precise estimation of causal effects using univariable MR. Other strengths include rigorous selection of genetic instruments that robustly and independently predicted the 12 RBC traits and examination of confounding and sensitivity analyses to identify pleiotropic violations of the exclusion‐restriction assumption using one of the largest biobanks globally with sufficient statistical power (Figure S5).

Some limitations of our study should be acknowledged. First, although hemoglobin is correlated with other RBC traits, our findings in the univariable MR are unlikely caused by pleiotropic effects of other RBC traits, as MR‐BMA suggests they do not appear to be causal or only have minor direct effects on VTE. Second, MR‐BMA is a statistical variable selection method; as is common for variable selection methods, it does not provide unbiased effect estimates. MR‐BMA effect estimates were shrunk toward the null because of accounting for selection across a large number of traits. Bias in the effect estimates is traded for reduced variance to stabilize and improve the selection of causal risk factors from several RBC traits. Instead, we provided unbiased estimates using standard univariable MR. Third, the hematological GWAS implemented high‐quality procedures to maximize the precision of blood cell traits, but complete measurement accuracy is impossible. A more accurately measured trait would inevitably be prioritized over a less accurately measured trait; we cannot exclude the possibility that hemoglobin is easier to measure accurately than hematocrit. Fourth, although ≈30% of participants overlapped, with strong instruments (explaining 4.2% of the variance in hemoglobin, with F statistic of 93; Figure S6), bias caused by sample overlap is likely to be negligible (bias, 0.004).^30^ Fifth, we cannot rule out selection bias in the UK Biobank and the INTERVAL study, resulting from the recruitment of generally healthier participants and survivors, which might bias toward the null.^37^ Sixth, because of the lack of sex‐specific GWAS of RBC traits, we did not assess the sex‐specific associations of hemoglobin with VTE, although the reference range for hemoglobin^38^ and the VTE incident rate^39^ are higher in men. So, our findings may go some way toward explaining higher VTE rates in men than women. Seventh, use of summary statistics precluded examination of nonlinear association; examination of threshold effects, when possible, would be clinically relevant. Eighth, 23% (40 521/173 480) of participants in the studies providing genetic associations with RBC traits were healthy blood donors from the INTERVAL study, but these GWASs did not adjust for blood donation frequency, which may impair precision of these estimates. Ninth, our estimates represent average causal effects in the general population, so they may not apply to all subgroups or translate into the optimal level of hemoglobin in at‐risk populations. Finally, our study compares groups of people with genetically predicted lower and higher hemoglobin to infer the effects of raising hemoglobin via ESAs and/or blood transfusion. However, several qualitative and quantitative differences between these comparisons may limit the applicability to intervening on hemoglobin. Specifically, small but lifelong changes in endogenous hemoglobin were determined by the genetic variants, via modulating a particular biological pathway, compared with large changes in hemoglobin within a short time.^32^

From a public health and clinical perspective, this study draws attention to factors that modulate hemoglobin, as potential targets of intervention to prevent thromboembolic events. This may include therapeutic phlebotomy^40^ and angiotensin II blockage.^41^ In contrast, testosterone induces erythrocytosis and substantially increases hemoglobin.^8^ Our findings may provide a potential mechanisms by which testosterone could increase the risk of VTE.^9^

In conclusion, the present MR study suggests hemoglobin could be the trait most relevant to VTE, and suggests a detrimental impact on VTE in the general population. Whether other factors that drive hemoglobin could be targets of intervention might bear consideration.

## Sources of Funding

This work was supported by the Small Project Funding from the University of Hong Kong (grant 201409176231 to Dr Au Yeung); Dr Luo is supported by the Bau Tsu Zung Bau Kwan Yeun Hing Research and Clinical Fellowship from the University of Hong Kong (grant *200008682.920006.20006.400.01); Drs Burgess and Zuber are supported by Sir Henry Dale fellowship, jointly funded by the Wellcome Trust and the Royal Society (grant 204623/Z/16/Z). The funders have no role in study design, data collection and analysis, decision to publish, or preparation of the manuscript.

## Disclosures

None.

**Figure 1:**
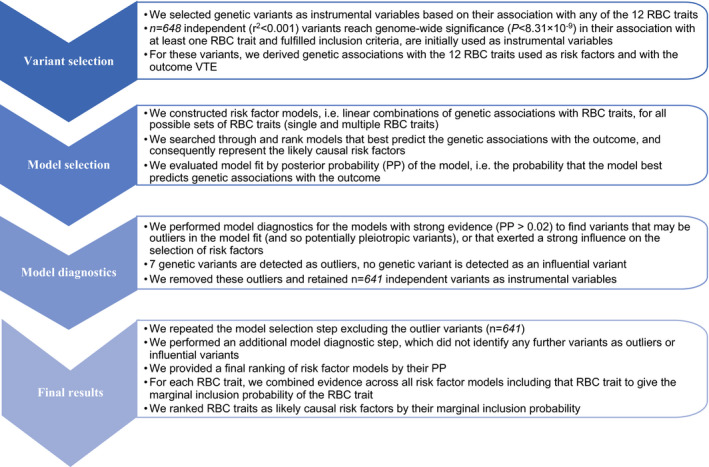
The arrow diagram of the multivariable mendelian randomization based on bayesian model averaging. PP indicates posterior probability; RBC, red blood cell; and VTE, venous thromboembolism.

## Supporting information


**Tables S1–S8**
**Figures S1–S6**
