# Supplementary material for: Impact of Genetically Predicted Red Blood Cell Traits on Venous Thromboembolism: Multivariable Mendelian Randomization Study Using UK Biobank
Source: J Am Heart Assoc. 2020 Jul 8;9(14):e016771. doi: 10.1161/JAHA.120.016771 (PMC7660720; doi:10.1161/JAHA.120.016771)
Supplement: Supplementary file 1 — Tables S1–S8 Figures S1–S6 [file JAH3-9-e016771-s001.pdf]

SUPPLEMENTAL MATERIAL

Table S1. Ranking of red blood cell traits for venous thromboembolism in the UK Biobank top panel) ranking of exposures according to their marginal inclusion probability and bottom panel) ranking of models (i.e. sets of exposures) according to their posterior probability

Ranking of exposures

	Exposure	Marginal inclusion probability	Model-averaged causal estimate (OR)
1	HGB	0.90	1.21
2	HCT	0.24	0.96
3	HLSR	0.18	0.98
4	RET%	0.11	0.99
5	MCHC	0.09	1.01
6	RBC	0.09	1.00
7	MCH	0.08	1.01
8	HLSR%	0.07	1.00
9	RET	0.07	1.00
10	IRF	0.07	1.01

Ranking of models (i.e. sets of exposures)

	Exposure(s)	Posterior probability	Model-specific causal estimate (OR)
1	HGB	0.45	1.16
2	HCT, HGB	0.07	0.82, 1.39
3	HGB, HLSR	0.04	1.20, 0.94
4	HGB, RBC	0.03	1.21, 0.94
5	HCT, HGB, HLSR	0.03	0.76, 1.53, 0.92
6	HGB, MCH	0.02	1.14, 1.04
7	MCH, RBC	0.02	1.15, 1.16
8	HCT, HGB, RET%	0.02	0.74, 1.56, 0.92
9	HGB, MCHC	0.02	1.14, 1.07
10	HGB, HLSR, RET%	0.01	1.25, 0.70, 1.32

HGB, haemoglobin concentration; HCT, haematocrit; MCHC, mean corpuscular haemoglobin concentration; HLSR, high light scatter reticulocyte count; RBC, red blood cell count; MCH, mean corpuscular haemoglobin; RET%, reticulocyte fraction of red cells; RET, reticulocyte count; HLSR%, high light scatter reticulocyte fraction of red cells; MCV, mean corpuscular volume; IRF, immature fraction of reticulocytes; RDW, red cell distribution width. Calculation is based on 648 genetic variants, using $\sigma^2 = 0.25$ as prior variance and $p = 0.1$ as prior probability, corresponding to *a priori* expecting one causal factor.

Table S2. Q statistics using n = 648 genetic variants for the best individual models and the maximum Q of each variant among these models for diagnostics.

No	Variant	Gene	Q M1	Q M2	Q M3	Q M4	Q M5	max Q
1	rs77542162	ABCA6	**20.79**	**20.82**	**20.43**	**20.34**	**20.36**	**20.82**
2	rs174533	MYRF	**16.28**	**16.31**	**15.94**	**15.72**	**15.88**	**16.31**
3	rs11187938	TBC1D12	**15.92**	**15.90**	**15.40**	**15.69**	**15.22**	**15.92**
4	rs738408	PNPLA3	**13.94**	**14.09**	**14.08**	**14.17**	**14.33**	**14.33**
5	rs3747207	PNPLA3	**13.49**	**13.67**	**13.63**	**13.73**	**13.91**	**13.91**
6	rs139974673	CATSPER2P1	**11.39**	**11.15**	**11.19**	**11.15**	**10.79**	**11.39**
7	rs147233090	CATSPER2P1	**11.00**	**10.76**	**10.77**	**10.77**	**10.39**	**11.00**
8	rs78378222	TP53	7.99	8.29	8.37	8.44	8.89	8.89
9	rs11122449	GALNT2	8.74	8.58	8.71	8.75	8.48	8.75
10	rs41282676	EIF2AK1	7.49	7.44	8.42	7.45	8.63	8.63
11	rs2835349	AP000695.6	8.42	7.91	8.43	8.23	7.74	8.43
12	rs35979828	NFE2	8.08	8.34	7.90	7.68	8.20	8.34
13	rs9535495	DLEU7	7.71	7.83	7.04	7.69	7.00	7.83
14	rs17248895	PLEK2	7.29	7.36	7.53	7.20	7.69	7.69
15	rs1339847	TRIM58	7.65	7.34	5.16	7.69	4.19	7.69
16	rs4859682	SHROOM3	7.34	6.85	7.46	7.06	6.83	7.46
17	rs6880621	CTD-2197M16.1	6.71	7.12	6.65	6.83	7.19	7.19
18	rs972761	CTD-2197M16.1	6.45	6.83	6.39	6.55	6.90	6.90
19	rs6712203	COBLL1	6.44	6.73	5.99	6.43	6.22	6.73
20	rs4434553	TFR2	6.08	5.10	6.67	5.13	5.45	6.67
21	rs61750953	EGLN2	6.27	6.55	5.99	6.33	6.28	6.55
22	rs73652622	MIR4289	6.01	6.55	5.37	5.93	5.88	6.55
23	rs78415359	CTIF	6.16	6.27	6.17	6.37	6.33	6.37
24	rs833805	RP5-1120P11.1	5.74	6.32	5.51	5.99	6.20	6.32
25	rs72996113	RN7SL222P	5.67	6.12	5.01	5.56	5.39	6.12
26	rs13389219	COBLL1	5.82	6.07	5.40	5.79	5.61	6.07
27	rs964184	ZNF259	5.51	5.02	6.07	5.57	5.53	6.07
28	rs12548939	PVT1	5.89	5.73	5.99	5.51	5.79	5.99
29	rs1569419	PRDM16	5.97	5.68	5.99	5.55	5.60	5.99
30	rs56235845	RGS14	5.02	5.28	5.08	5.15	5.46	5.46

Q, Q statistics; M: Model. M1 (HGB), M2 (HCT and HGB), M3 (HGB and HLSR), M4 (HGB and RBC), M5 (HCT, HGB and HLSR). Variants with Q statistics > 10 are given in bold. This table displays the 30 variants with the largest maximum Q and the gene region they fall in.

Table S3. Cook's distance using n = 648 genetic variants for the best individual models and the maximum Cook's distance of each variant among these models for diagnostics.

	Variant	Gene	Cd M1	Cd M2	Cd M3	Cd M4	Cd M5	max Cd
1	rs77542162	ABCA6	0.079	0.04	0.041	0.042	0.027	0.079
2	rs1339847	TRIM58	0	0.002	0.069	0	0.044	0.069
3	rs73728279	PRKAG2	0.05	0.028	0.027	0.025	0.019	0.05
4	rs10224210	PRKAG2	0.049	0.027	0.027	0.024	0.019	0.049
5	rs198851	HIST1H1T	0.035	0.043	0.017	0.046	0.031	0.046
6	rs1799945	HFE	0.034	0.042	0.016	0.045	0.031	0.045
7	rs174533	MYRF	0.04	0.02	0.021	0.024	0.014	0.04
8	rs833805	RP5-1120P11.1	0.034	0.025	0.017	0.019	0.016	0.034
9	rs10168349	PRKCE	0.03	0.014	0.017	0.014	0.01	0.03
10	rs738408	PNPLA3	0.028	0.015	0.015	0.015	0.01	0.028
11	rs10495928	PRKCE	0.028	0.013	0.016	0.013	0.009	0.028
12	rs147233090	CATSPER2P1	0.027	0.014	0.014	0.014	0.01	0.027
13	rs3747207	PNPLA3	0.027	0.014	0.014	0.015	0.01	0.027
14	rs2968478	PIEZO1	0.026	0.019	0.016	0.013	0.014	0.026
15	rs139974673	CATSPER2P1	0.024	0.013	0.012	0.013	0.009	0.024
16	rs2106786	SPPL2C	0.022	0.014	0.012	0.014	0.012	0.022
17	rs17563683	LINC02210-CRHR1	0.021	0.013	0.012	0.013	0.011	0.021
18	rs4606752	KANSL1	0.02	0.013	0.012	0.013	0.012	0.02
19	rs4434553	TFR2	0.012	0.019	0.011	0.017	0.015	0.019
20	rs2923411	RP11-503E24.3	0.01	0.017	0.005	0.005	0.011	0.017
21	rs551238	EPO	0.01	0.016	0.006	0.014	0.011	0.016
22	rs2835349	AP000695.6	0.016	0.012	0.008	0.008	0.008	0.016
23	rs11970772	CCND3	0	0	0.005	0.016	0.004	0.016
24	rs12548939	PVT1	0.014	0.007	0.007	0.009	0.005	0.014
25	rs972761	CTD-2197M16.1	0.014	0.01	0.007	0.007	0.007	0.014
26	rs6880621	CTD-2197M16.1	0.013	0.01	0.007	0.007	0.007	0.013
27	rs837763	PIEZO1	0.013	0.009	0.008	0.007	0.006	0.013
28	rs34164109	HBS1L	0.012	0.013	0.009	0.013	0.008	0.013
29	rs72805692	HK1	0.009	0.009	0.008	0.004	0.013	0.013
30	rs592423	AL356739.1	0.002	0.001	0.009	0.013	0.005	0.013
	threshold		0.455	0.694	0.694	0.694	0.789	

Cd Cook distance; M: Model. M1 (HGB), M2 (HCT and HGB), M3 (HGB and HLSR), M4 (HGB and RBC), M5 (HCT, HGB and HLSR). The final line gives the suggested cut-off for Cook's distance. This table displays the 30 variants with the largest maximum Cook's distance and the gene region they fall in.

Table S4. Q statistics using n = 641 genetic variants after exclusion of outlying variants, for the best individual models and the maximum Q of each variant among these models for diagnostics.

No	Variant	Gene	Q M1	Q M2	Q M3	Q M4	Q M5	max Q
1	rs78378222	TP53	8.00	8.29	8.34	8.85	8.40	8.85
2	rs11122449	GALNT2	8.75	8.59	8.72	8.49	8.76	8.76
3	rs41282676	EIF2AK1	7.50	7.44	8.34	8.54	7.45	8.54
4	rs2835349	AP000695.6	8.40	7.90	8.42	7.75	8.24	8.42
5	rs35979828	NFE2	8.08	8.33	7.92	8.20	7.72	8.33
6	rs9535495	DLEU7	7.72	7.83	7.11	7.06	7.70	7.83
7	rs1339847	TRIM58	7.65	7.35	5.36	4.40	7.69	7.69
8	rs17248895	PLEK2	7.30	7.36	7.51	7.67	7.21	7.67
9	rs4859682	SHROOM3	7.33	6.84	7.44	6.83	7.08	7.44
10	rs6880621	CTD-2197M16.1	6.72	7.13	6.67	7.19	6.83	7.19
11	rs972761	CTD-2197M16.1	6.46	6.84	6.41	6.90	6.55	6.90
12	rs6712203	COBLL1	6.43	6.71	6.02	6.25	6.42	6.71
13	rs4434553	TFR2	6.07	5.10	6.60	5.42	5.20	6.60
14	rs61750953	EGLN2	6.29	6.56	6.03	6.31	6.34	6.56
15	rs73652622	MIR4289	6.01	6.54	5.42	5.92	5.93	6.54
16	rs78415359	CTIF	6.17	6.28	6.18	6.33	6.36	6.36
17	rs833805	RP5-1120P11.1	5.77	6.33	5.55	6.22	5.99	6.33
18	rs72996113	RN7SL222P	5.67	6.12	5.07	5.44	5.57	6.12
19	rs13389219	COBLL1	5.81	6.06	5.43	5.63	5.78	6.06
20	rs964184	ZNF259	5.51	5.02	6.02	5.50	5.57	6.02
21	rs12548939	PVT1	5.91	5.74	5.99	5.79	5.55	5.99
22	rs1569419	PRDM16	5.97	5.68	5.98	5.61	5.59	5.98
23	rs56235845	RGS14	5.03	5.28	5.09	5.45	5.14	5.45
24	rs17006441	MITF	5.28	5.03	5.38	5.08	5.11	5.38
25	rs2106786	SPPL2C	4.52	4.89	4.73	5.31	4.87	5.31
26	rs17563683	LINC02210-CRHR1	4.45	4.80	4.68	5.25	4.76	5.25
27	rs159058	NOL4L	4.85	4.95	4.65	4.72	5.24	5.24
28	rs4606752	KANSL1	4.36	4.73	4.62	5.22	4.69	5.22
29	rs717662	RN7SL222P	4.72	5.13	4.20	4.54	4.65	5.13
30	rs3812049	SLC12A2	4.95	5.09	4.91	5.09	4.93	5.09

Q, Q statistics; M: model. M1 (HGB), M2 (HCT and HGB), M3 (HGB and HLSR), M4 (HCT, HGB and HLSR), M5 (HGB and MCHC). This table displays the 30 variants with the largest maximum Q and the gene region they fall in.

Table S5. Cook's distance using n = 641 genetic variants after exclusion of outlying variants, the best individual models and the maximum Cook's distance of each variant among these models for diagnostics.

No	Variant	Gene	Cd M1	Cd M2	Cd M3	Cd M4	Cd M5	max Cd
1	rs1339847	TRIM58	0.001	0.002	0.08	0.052	0	0.08
2	rs73728279	PRKAG2	0.057	0.032	0.031	0.022	0.028	0.057
3	rs10224210	PRKAG2	0.056	0.031	0.03	0.021	0.027	0.056
4	rs198851	HIST1H1T	0.039	0.048	0.019	0.035	0.05	0.05
5	rs1799945	HFE	0.039	0.048	0.019	0.035	0.049	0.049
6	rs833805	RP5-1120P11.1	0.04	0.028	0.02	0.019	0.022	0.04
7	rs10168349	PRKCE	0.034	0.016	0.019	0.011	0.016	0.034
8	rs10495928	PRKCE	0.032	0.015	0.018	0.01	0.015	0.032
9	rs2968478	PIEZO1	0.03	0.022	0.018	0.016	0.015	0.03
10	rs2106786	SPPL2C	0.025	0.016	0.014	0.013	0.016	0.025
11	rs17563683	LINC02210-CRHR1	0.024	0.015	0.014	0.013	0.015	0.024
12	rs4606752	KANSL1	0.023	0.015	0.013	0.013	0.015	0.023
13	rs4434553	TFR2	0.013	0.022	0.013	0.016	0.02	0.022
14	rs2923411	RP11-503E24.3	0.011	0.019	0.006	0.012	0.006	0.019
15	rs551238	EPO	0.011	0.019	0.007	0.012	0.016	0.019
16	rs2835349	AP000695.6	0.018	0.013	0.009	0.009	0.01	0.018
17	rs11970772	CCND3	0	0	0.006	0.004	0.017	0.017
18	rs34164109	HBS1L	0.014	0.014	0.01	0.009	0.017	0.017
19	rs12548939	PVT1	0.016	0.008	0.008	0.006	0.01	0.016
20	rs972761	CTD-2197M16.1	0.016	0.011	0.008	0.008	0.008	0.016
21	rs6880621	CTD-2197M16.1	0.015	0.011	0.008	0.008	0.008	0.015
22	rs9376090	HBS1L	0.012	0.013	0.008	0.008	0.015	0.015
23	rs837763	PIEZO1	0.015	0.01	0.009	0.007	0.008	0.015
24	rs4859682	SHROOM3	0.014	0.011	0.008	0.007	0.008	0.014
25	rs72805692	HK1	0.011	0.01	0.009	0.014	0.005	0.014
26	rs5758896	A4GALT	0.014	0.007	0.007	0.005	0.008	0.014
27	rs592423	AL356739.1	0.002	0.001	0.01	0.006	0.014	0.014
28	rs7541039	PROX1	0.014	0.01	0.007	0.007	0.007	0.014
29	rs12548864	PVT1	0.013	0.007	0.007	0.005	0.008	0.013
30	rs41282676	EIF2AK1	0.001	0	0.013	0.01	0	0.013
	threshold		0.455	0.694	0.694	0.790	0.694	

CD Cook distance; M: model. M1 (HGB), M2 (HCT and HGB), M3 (HGB and HLSR), M4 (HCT, HGB and HLSR), M5 (HGB and MCHC). The final line gives the suggested cut-off for Cook's distance. This table displays the 30 variants with the largest maximum Cook's distance and the gene region they fall in.

Table S6. Parameter check for the prior probability p, ranging from p=0.2 to 0.4.

p = 0.2			
No.	Exposure	Marginal inclusion probability	model-averaged causal effect (OR)
1	HGB	0.874	1.32
2	HCT	0.539	0.88
3	HLSR	0.306	0.96
4	IRF	0.298	1.05
5	RET%	0.260	0.97
6	HLSR%	0.244	1.00
7	MCHC	0.231	1.03
8	RET	0.223	0.99
9	RBC	0.087	1.00
10	MCH	0.078	1.01
p = 0.3			
No.	Exposure	Marginal inclusion probability	model-averaged causal effect (OR)
1	HGB	0.855	1.38
2	HCT	0.660	0.85
3	IRF	0.451	1.08
4	HLSR	0.368	0.95
5	HLSR%	0.363	0.99
6	RET%	0.347	0.95
7	RET	0.335	0.99
8	MCHC	0.305	1.04
9	RBC	0.105	1.00
10	MCH	0.093	1.01
p = 0.4			
No.	Exposure	Marginal inclusion probability	model-averaged causal effect (OR)
1	HGB	0.842	1.40
2	HCT	0.706	0.83
3	IRF	0.541	1.11
4	HLSR%	0.445	0.98
5	RET	0.415	0.98
6	HLSR	0.408	0.96
7	RET%	0.403	0.95
8	MCHC	0.352	1.04
9	RBC	0.145	1.01
10	MCH	0.130	1.02

p = 0.2 to 0.4 reflects 2.4 to 4.8 expected causal exposures. OR, odds, ratio.

Table S7. Ranking of blood cell traits for venous thromboembolism with different selections of exposures, according to their marginal inclusion probability.

Cell lineage: 12 red blood cell and 4 platelet traits, n = 961

No.	Exposure	Marginal inclusion probability	model-averaged causal effect (OR)
1	HGB	0.915	1.22
2	HCT	0.211	0.96
3	HLSR	0.146	0.98
4	RBC	0.124	1.00
5	MCH	0.101	1.01
6	IRF	0.088	1.02
7	RET%	0.080	1.00
8	HLSR%	0.076	1.00
9	RET	0.071	0.99
10	MCHC	0.055	1.01

Cell lineage: 11 red blood cell traits (Exclude hematocrit), n = 578

No.	Exposure	Marginal inclusion probability	model-averaged causal effect (OR)
1	HGB	0.527	1.07
2	MCHC	0.329	1.04
3	HLSR	0.189	0.98
4	RET%	0.176	0.98
5	MCH	0.160	1.01
6	HLSR%	0.103	1.00
7	RET	0.092	1.00
8	IRF	0.089	1.01
9	RBC	0.071	1.00
10	MCV	0.061	1.00

Cell lineage: Red blood cell traits (Exclude hemoglobin), n = 566

No.	Exposure	Marginal inclusion probability	model-averaged causal effect (OR)
1	MCH	0.436	1.04
2	MCHC	0.343	1.04
3	HCT	0.177	1.02
4	RET%	0.158	0.97
5	RBC	0.154	1.02
6	MCV	0.150	1.01
7	HLSR	0.125	1.00
8	HLSR%	0.065	1.00
9	RET	0.064	1.01
10	IRF	0.048	1.01

OR, odds ratio. We used p = 0.1 as prior probability and excluded outlying variants in the above analyses.

Table S8. Genetic variants predicting haemoglobin concentration used in the univariable Mendelian randomization analyses.

						Haemoglobin		VTE			
Variant	Info	EA	OA	EAF	R^2 (%)	Beta	SE	Beta	SE	Exclude	PhenoScanner
rs1010269	0.98	A	G	0.17	0.028	0.031	0.005	0.014	0.020		
rs10495928	1.00	A	G	0.66	0.240	0.073	0.004	-0.009	0.015		
rs10899133	1.00	T	C	0.11	0.025	0.036	0.006	0.003	0.024		
rs11072567	1.00	A	G	0.49	0.066	0.036	0.004	0.000	0.015		
rs11122272	1.00	G	A	0.63	0.033	0.027	0.004	0.019	0.015		
rs115986297	1.00	A	G	0.46	0.038	0.028	0.004	0.005	0.015		
rs11749327	0.98	A	C	0.31	0.022	0.023	0.004	0.012	0.016		
rs11772705	1.00	C	T	0.29	0.036	0.030	0.004	0.004	0.016		
rs1181870	0.95	A	C	0.24	0.037	0.032	0.004	-0.019	0.018		
rs1182933	1.00	T	C	0.30	0.024	0.024	0.004	-0.027	0.016	√	CHD, cholesterol
rs123698	1.00	C	G	0.60	0.036	0.027	0.004	-0.017	0.015		
rs12548874	1.00	C	A	0.53	0.021	0.021	0.004	-0.019	0.015		
rs1256061	1.00	G	T	0.52	0.030	0.024	0.004	0.023	0.015		
rs12811512	1.00	C	T	0.85	0.021	0.029	0.005	0.006	0.020		
rs128494	0.97	C	T	0.77	0.036	0.032	0.004	-0.018	0.017		
rs12945870	1.00	C	T	0.43	0.026	0.023	0.004	0.030	0.015		
rs1340818	0.99	C	T	0.61	0.025	0.023	0.004	-0.002	0.015		
rs144861591	0.98	T	C	0.08	0.466	0.181	0.007	0.033	0.027		
rs147233090	0.99	C	T	0.98	0.041	0.093	0.012	0.181	0.050		
rs17006441	0.99	A	C	0.42	0.025	0.023	0.004	-0.031	0.015		
rs174533	1.00	A	G	0.35	0.036	0.028	0.004	-0.058	0.015	√	Cholesterol
rs17476364	1.00	C	T	0.11	0.450	0.151	0.006	0.026	0.023		
rs17563683	1.00	G	A	0.23	0.068	0.043	0.004	0.043	0.017	√	Blood pressure
rs17773190	0.98	G	A	0.48	0.028	0.024	0.004	0.021	0.015		
rs184088518	0.93	G	T	0.98	0.047	0.101	0.012	-0.004	0.048		
rs1997595	0.98	A	C	0.66	0.030	0.031	0.004	0.002	0.015		
rs218264	0.98	A	T	0.75	0.044	0.034	0.004	0.014	0.017		
rs2186037	1.00	G	A	0.52	0.044	0.024	0.004	0.003	0.015		
rs2230657	1.00	G	A	0.53	0.033	0.026	0.004	0.004	0.015		
rs2246363	0.99	G	A	0.25	0.023	0.025	0.004	-0.004	0.017		
rs2269188	0.94	G	C	0.72	0.031	0.028	0.004	0.014	0.017		
rs228917	1.00	T	C	0.57	0.025	0.044	0.004	0.007	0.015		
rs2519796	0.99	G	A	0.33	0.021	0.024	0.004	-0.006	0.016		
rs261332	1.00	G	A	0.79	0.031	0.025	0.004	0.030	0.018	√	Cholesterol
rs2870238	1.00	T	C	0.50	0.024	0.025	0.004	-0.014	0.015		
rs2878889	0.99	A	G	0.55	0.031	0.022	0.004	0.000	0.015		
rs2923411	1.00	C	T	0.59	0.031	0.025	0.004	0.036	0.015		
rs2928166	0.99	C	T	0.13	0.127	0.037	0.005	0.029	0.022		
rs2968478	0.96	T	G	0.42	0.024	0.051	0.004	0.034	0.015		
rs35060063	1.00	G	A	0.50	0.023	0.022	0.004	-0.006	0.015		
rs3811444	1.00	T	C	0.34	0.022	0.023	0.004	-0.025	0.016		
rs3996993	1.00	C	T	0.53	0.024	0.021	0.004	-0.022	0.015		
rs4073770	0.99	A	T	0.75	0.046	0.025	0.004	0.001	0.017		
rs442177	1.00	G	T	0.41	0.023	0.031	0.004	0.030	0.015	√	Cholesterol

rs447735	1.00	C	T	0.42	0.071	0.022	0.004	-0.002	0.015		
rs4760682	1.00	C	A	0.19	0.026	0.048	0.005	-0.007	0.019		
rs4791641	1.00	C	T	0.50	0.027	0.023	0.004	-0.009	0.015	√	Cholesterol
rs4951074	0.99	A	G	0.10	0.021	0.039	0.006	0.044	0.024		
rs4957325	0.99	C	T	0.12	0.022	0.032	0.006	-0.016	0.023		
rs554019	1.00	C	T	0.58	0.023	0.021	0.004	0.017	0.015		
rs56235845	0.99	G	T	0.33	0.026	0.023	0.004	0.038	0.016		
rs56262900	0.99	A	G	0.10	0.096	0.037	0.006	0.016	0.024		
rs5758896	1.00	C	T	0.59	0.046	0.031	0.004	0.034	0.015		
rs57908212	0.99	C	T	0.47	0.037	0.027	0.004	0.012	0.015		
rs58017093	1.00	A	C	0.37	0.031	0.026	0.004	0.006	0.015		
rs59901009	1.00	T	C	0.76	0.088	0.049	0.004	0.029	0.017		
rs6064559	1.00	A	C	0.60	0.024	0.022	0.004	-0.003	0.015		
rs61750953	1.00	C	T	0.98	0.024	0.088	0.014	0.166	0.061		
rs62435145	0.94	G	T	0.31	0.036	0.029	0.004	-0.014	0.016		
rs6459467	1.00	G	A	0.62	0.025	0.023	0.004	-0.002	0.015		
rs662735	0.99	A	T	0.80	0.026	0.029	0.004	-0.012	0.018		
rs66561647	0.99	C	T	0.66	0.037	0.029	0.004	0.031	0.015		
rs6665764	1.00	A	G	0.26	0.047	0.035	0.004	0.036	0.016		
rs66782572	1.00	A	G	0.46	0.021	0.021	0.004	0.026	0.015		
rs67145503	0.99	A	T	0.12	0.050	0.049	0.006	0.043	0.023		
rs6841433	1.00	T	G	0.82	0.023	0.028	0.005	-0.018	0.019		
rs6967414	1.00	A	G	0.11	0.025	0.036	0.006	-0.041	0.024		
rs7045087	1.00	T	C	0.70	0.023	0.023	0.004	0.009	0.016		
rs73728279	0.99	G	T	0.72	0.167	0.064	0.004	-0.024	0.016		
rs753381	1.00	C	T	0.55	0.021	0.021	0.004	0.004	0.015	√	Cholesterol
rs7560180	0.98	T	A	0.22	0.047	0.037	0.004	0.013	0.018		
rs768090	0.99	A	T	0.32	0.027	0.025	0.004	-0.029	0.016		
rs77542162	1.00	G	A	0.02	0.044	0.099	0.012	0.213	0.043	√	Cholesterol
rs7875291	0.99	G	A	0.64	0.047	0.032	0.004	-0.005	0.015		
rs7945705	0.99	G	A	0.55	0.046	0.030	0.004	-0.017	0.015		
rs8055546	1.00	T	C	0.07	0.030	0.048	0.007	-0.031	0.029		
rs833805	0.91	G	A	0.89	0.098	0.070	0.006	0.069	0.024		
rs8887	0.98	C	T	0.57	0.034	0.027	0.004	-0.019	0.015		
rs9376090	1.00	T	C	0.74	0.095	0.050	0.004	-0.013	0.017	√	Cholesterol
rs9472135	0.99	T	C	0.69	0.056	0.036	0.004	0.000	0.016		
rs972761	0.98	T	C	0.53	0.032	0.025	0.004	0.041	0.015		

Beta (standard error) with haemoglobin concentration/ venous thromboembolism (VTE) are the changes in haemoglobin concentration (g/dL)/ log-transformed venous thromboembolism per additional copy of the effect allele; Estimates of variant on haemoglobin concentration are taken from Astle et al. Estimates of variant on venous thromboembolism are derived with individual data in the UK Biobank. EA, effect allele; OA, other allele; EAF, effect allele frequency; SE, standard error; R^2, the proportion of variance explained for the association between variant and haemoglobin concentration, presented in percentage. Exclude, a tick indicates the variant is associated with potential causes of venous thromboembolism ($P < 5\times10^{-8}$) based on PhenoScanner, excluded in the analysis. CHD, coronary heart disease

Figure S1. Directed acyclic graph of instrumental variable assumptions made in multivariable Mendelian randomization.

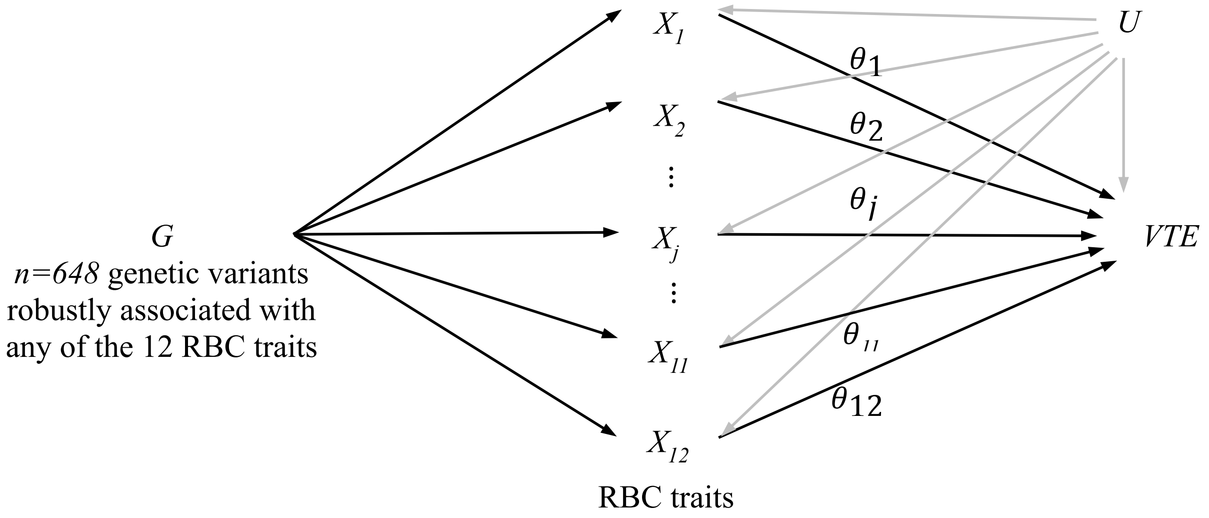

G = Genetic variants, X_j = risk factor j for j = 1, …,12 red blood cell traits, U = confounders, θ_j = causal effect of risk factor j for j = 1, …,12 red blood cell traits.

Figure S2. Genetic correlation between 12 red blood cell traits based on the n = 648 genetic variants used as instrumental variables.

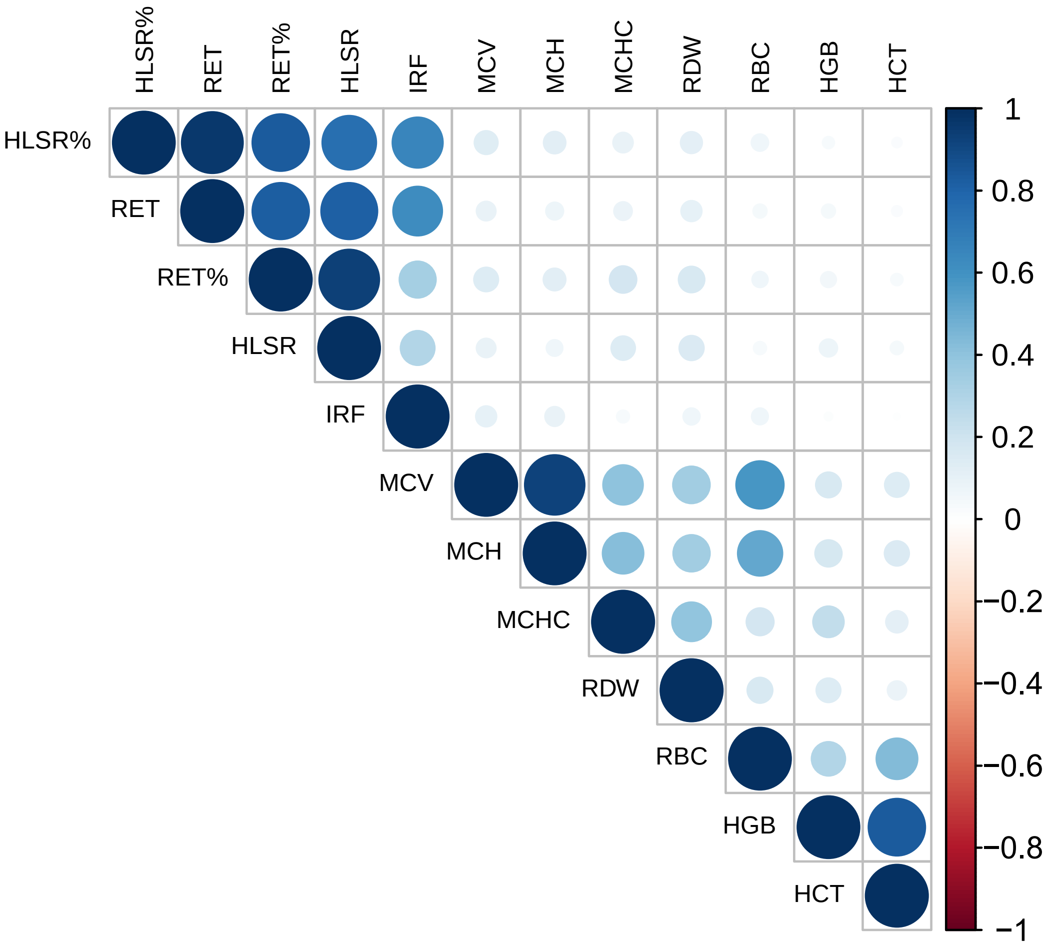

1) HGB

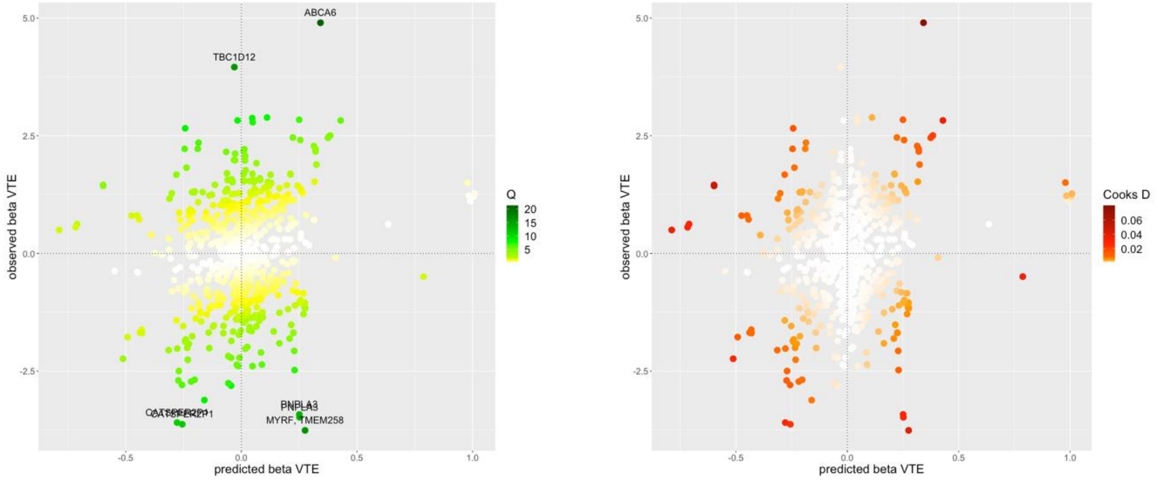

2) HCT and HGB

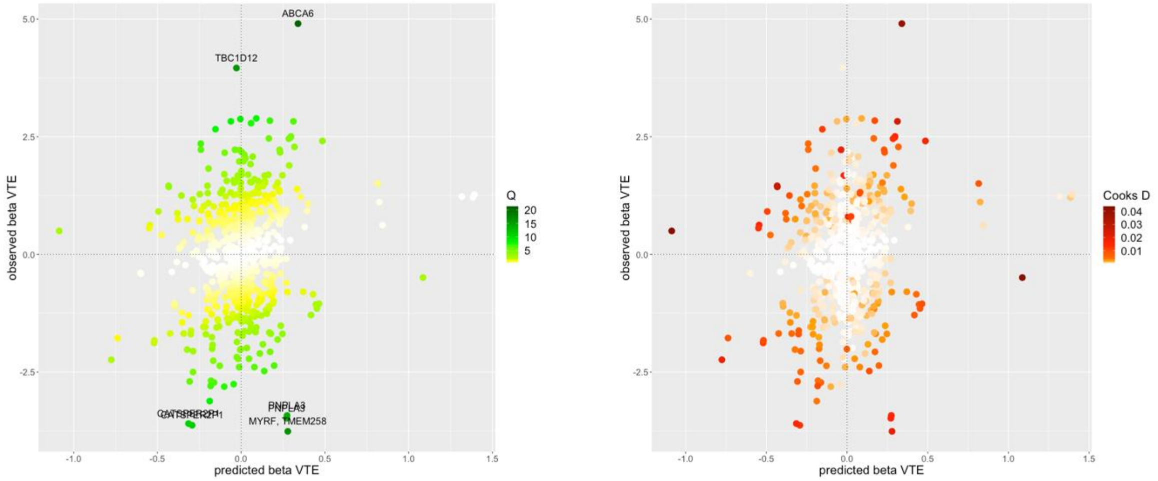

3) HGB and HLSR

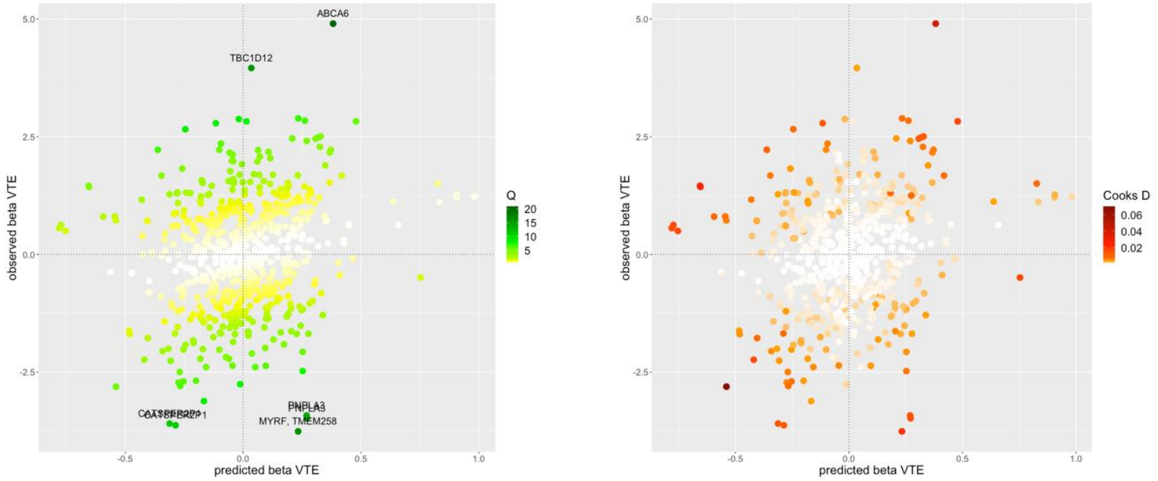

4) HGB and RBC

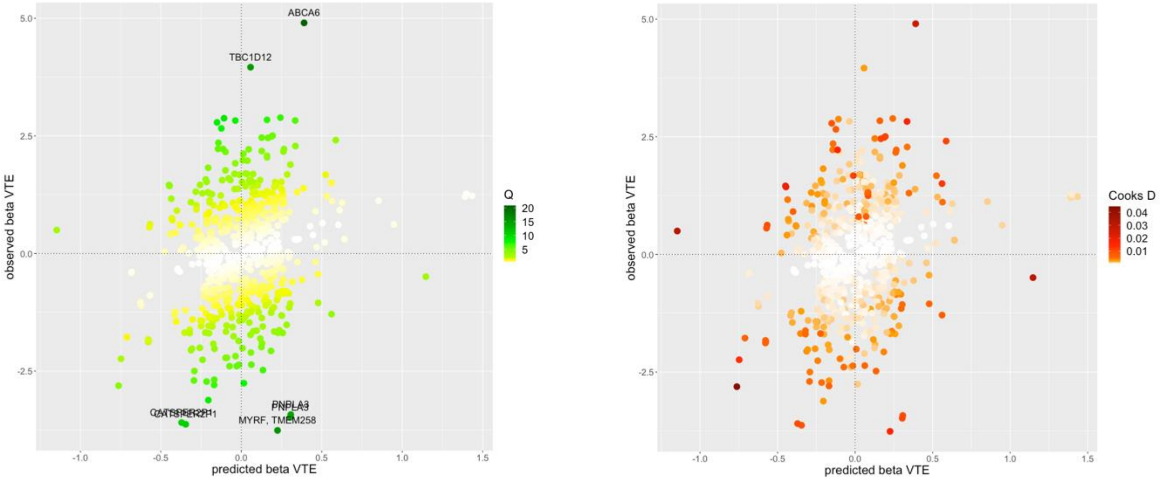

5) HCT, HGB and HLSR

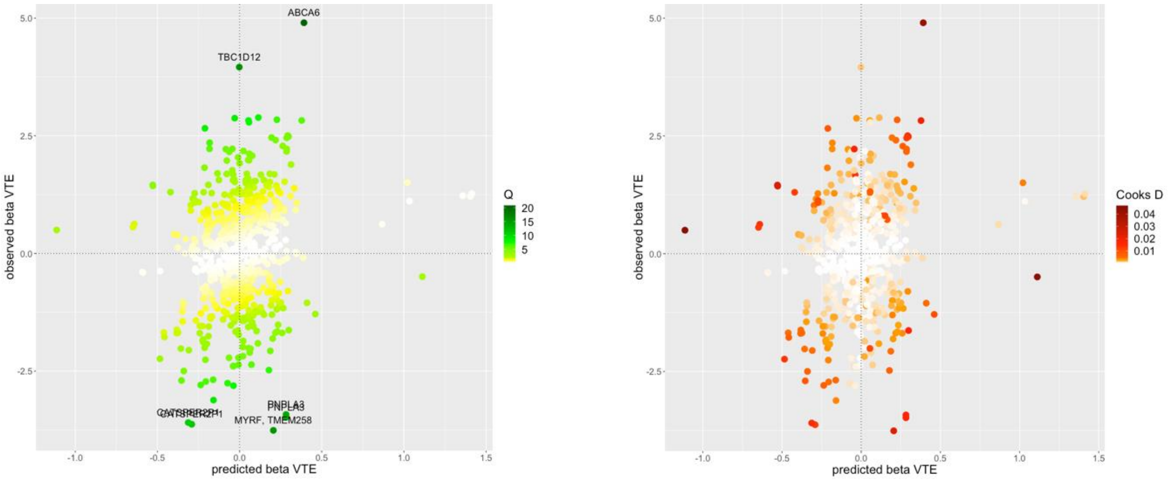

Figure S3. Diagnostic plots of the predicted associations with venous thromboembolism (VTE) (x-axis) based on the best individual models 1 (HGB), model 2, (HCT and HGB), model 3 (HGB and HLSR), model 4 (HGB and RBC), model 5 (HCT, HGB and HLSR), against the observed associations with VTE (y-axis). The colour code shows left) the Q-statistics for outliers and right) Cook's distance for influential points. Any genetic variant with Q-statistic larger than 10 or Cook's distance larger than median is marked by a label indicating the gene region.

Figure S4. Scatterplot of associations with A) haemoglobin and B) haematocrit on the x-axis against the association with venous thromboembolism (VTE) y-axis after excluding the outlying variants.

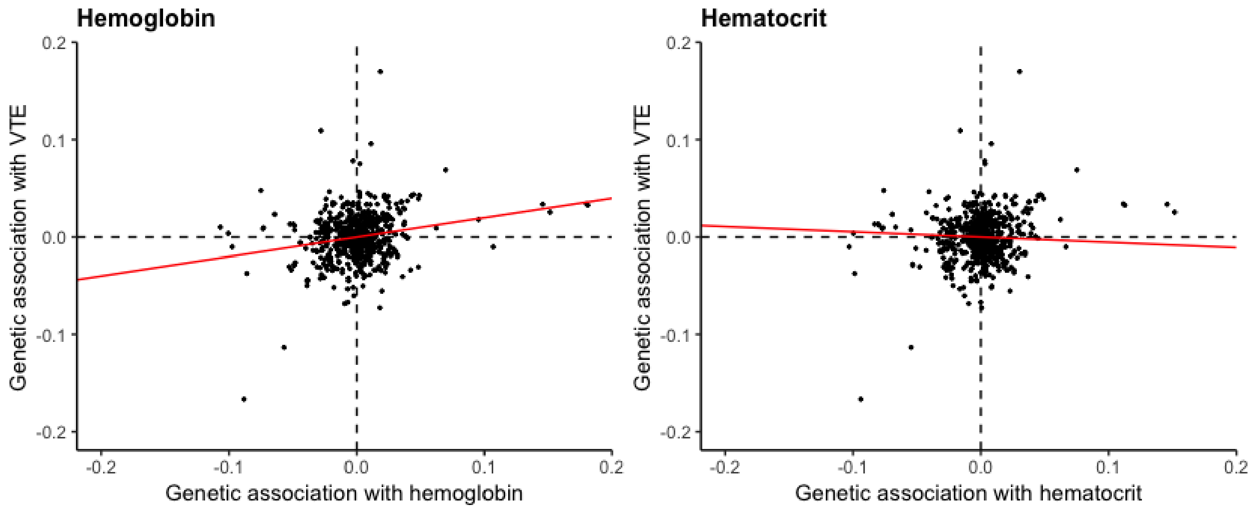

The mode-averaged causal effect of each exposure on VTE was marked in red.

1) HGB

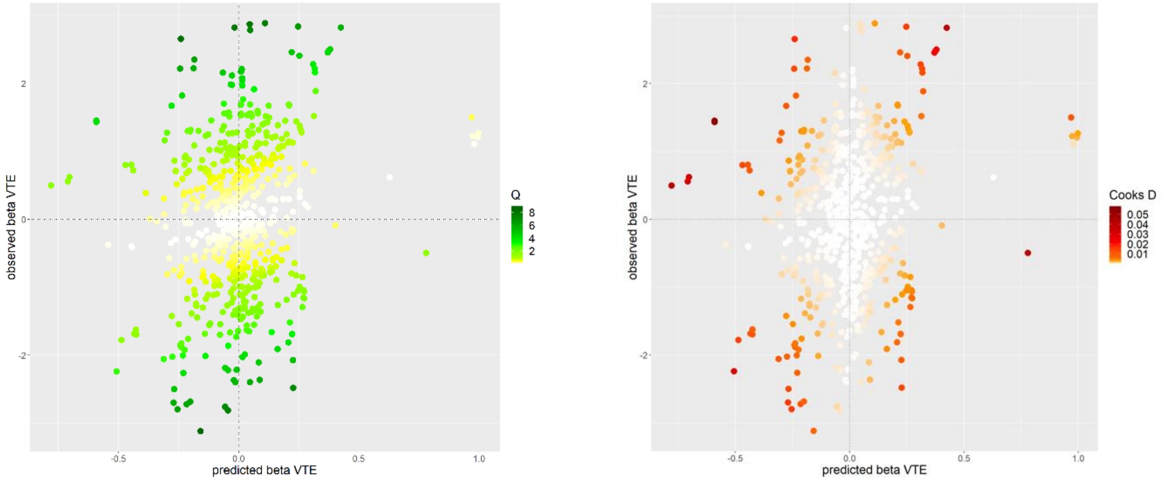

2) HCT and HGB

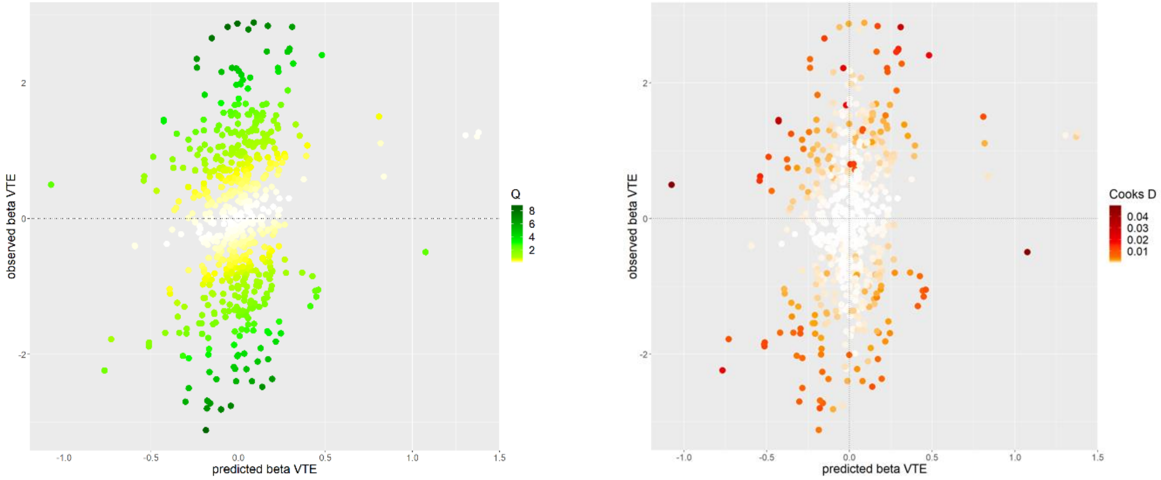

3) HGB and HLSR

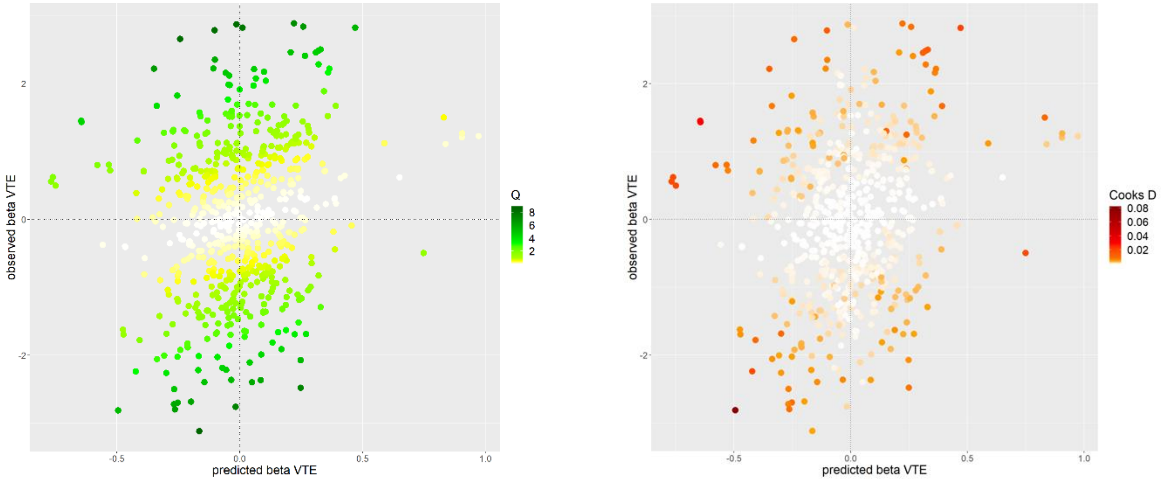

4) HCT, HGB and HLSR

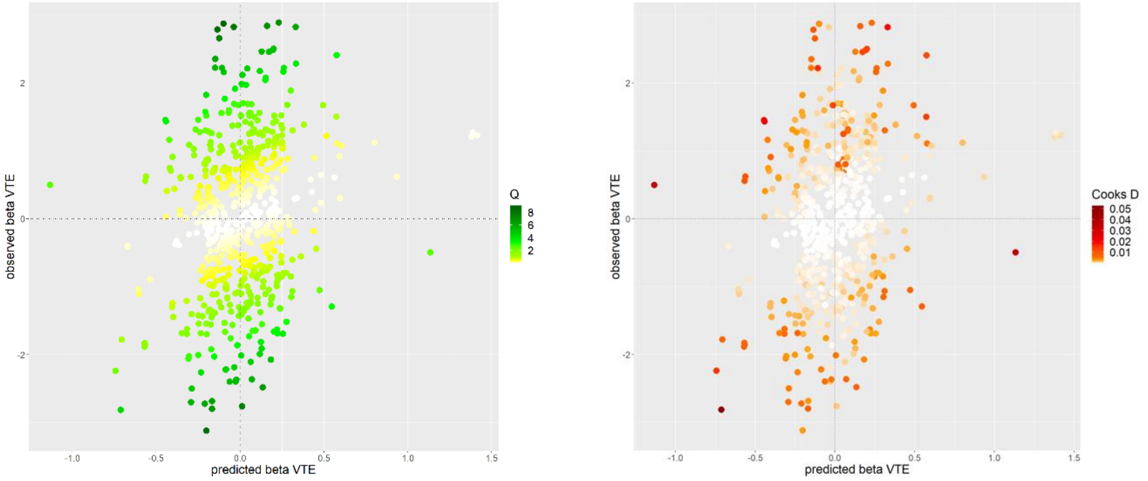

5) HGB and RBC

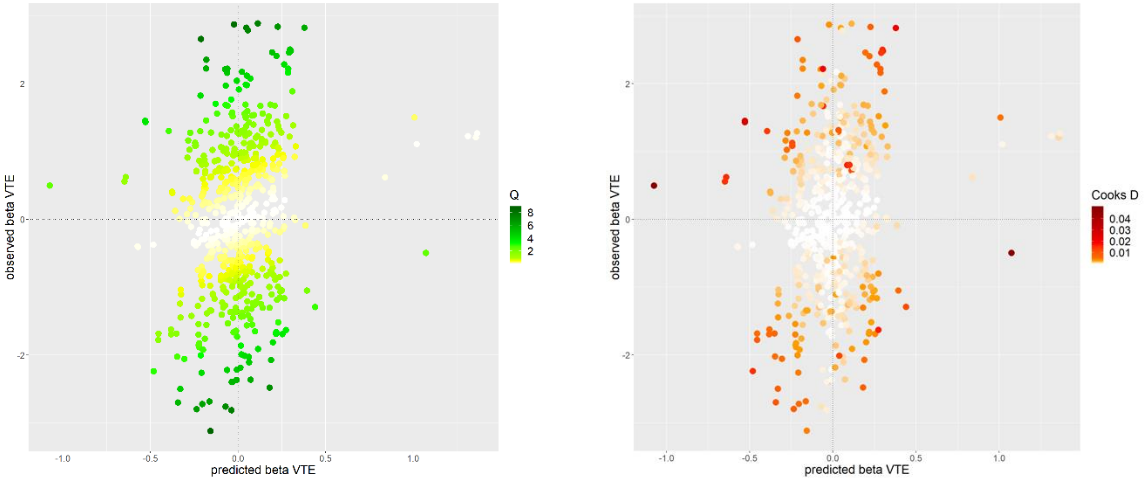

Figure S5. Diagnostic plots of the predicted associations with venous thromboembolism (VTE) (x-axis) based on the best individual models 1 (HGB), model 2, (HCT and HGB), model 3 (HGB and HLSR), model 4 (HCT, HGB and HLSR) and model 5 (HGB and RBC), against the observed associations with VTE (y-axis), after excluding the outlying variants. The colour code shows left) the Q-statistics for outliers and right) Cook's distance for influential points. Any genetic variant with Q-statistics large than 10 or Cook's distance large than median is marked by a label indicating the gene region.

Figure S6. Power curves for venous thromboembolism with a sample size of 265 424 with 9 752 venous thromboembolism cases in the UK Biobank.

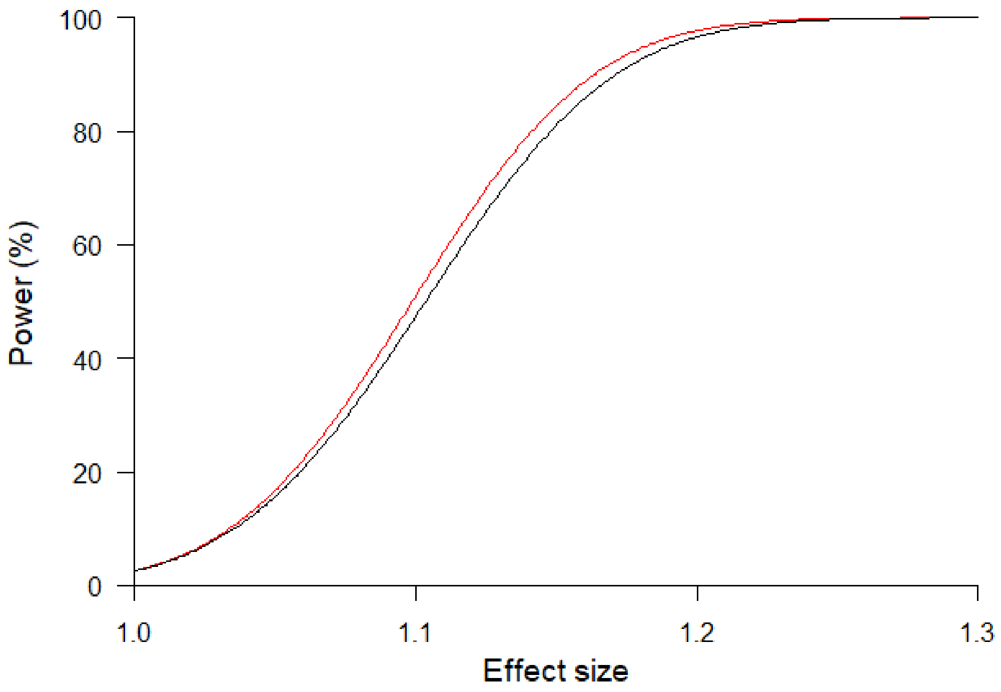

The red line represents power in the venous thromboembolism analysis using 81 genetic variants explaining 4.2% of the variance in haemoglobin, with F-statistics of 93; the black line represents power in the venous thromboembolism analysis using 72 genetic variants explaining 3.8% of the variance in haemoglobin, with F-statistics of 95. Power is reasonable (above 80%) for effect sizes of 1.14 and 1.15 for venous thromboembolism in the UK Biobank.
